# Development and Numerical Implementation of a Modified Mixed-Mode Traction–Separation Law for the Simulation of Interlaminar Fracture of Co-Consolidated Thermoplastic Laminates Considering the Effect of Fiber Bridging

**DOI:** 10.3390/ma15155108

**Published:** 2022-07-22

**Authors:** Ioannis Sioutis, Konstantinos Tserpes

**Affiliations:** Laboratory of Technology & Strength of Materials, Department of Mechanical Engineering & Aeronautics, University of Patras, 26500 Patras, Greece; sioutis.i@upnet.gr

**Keywords:** thermoplastics, co-consolidated joints, fracture toughness, cohesive zone modeling, finite element analysis

## Abstract

In the present work, a numerical model based on the cohesive zone modeling (CZM) approach has been developed to simulate mixed-mode fracture of co-consolidated low melt polyaryletherketone thermoplastic laminates by considering fiber bridging. A modified traction separation law of a tri-linear form has been developed by superimposing the bi-linear behaviors of the matrix and fibers. Initially, the data from mode I (DCB) and mode II (ENF) fracture toughness tests were used to construct the R-curves of the joints in the opening and sliding directions. The constructed curves were incorporated into the numerical models employing a user-defined material subroutine developed in the LS-Dyna finite element (FE) code. A numerical method was used to extract the fiber bridging law directly from the simulation results, thus eliminating the need for the continuous monitoring of crack opening displacement during testing. The final cohesive model was implemented via two identical FE models to simulate the fracture of a Single-Lap-Shear specimen, in which a considerable amount of fiber bridging was observed on the fracture area. The numerical results showed that the developed model presented improved accuracy in comparison to the CZM with the bi-linear traction–separation law (T–SL) in terms of the predicted strength of the joint.

## 1. Introduction

Polymeric matrix composite materials have become the norm for the aerospace industry in the past few decades. Various factors led to this transition over their metallic counterparts, such as their high strength-to-weight ratio and application dependent properties’ designation [1]. The multi-material composition of fiber reinforced polymers has led to the emergence of numerous mechanical phenomena, inducing complexity in the study of their fracture behavior.

Fiber bridging constitutes such a phenomenon that has been under investigation and impacts the delamination behavior of composite materials. Generally, fiber bridging is defined as the separation of fibers or fiber bundles during the debonding of adjacent plies, thus increasing the interlaminar fracture toughness. The severity of this phenomenon is attributed by factors such as fiber orientation and matrix–fiber interface strength [2]. Numerous works have been published, focusing on both the definition of the mechanical behavior and the usage of fiber bridging in favor of the structure’s integrity through thickness reinforcement. Available studies include analytical approaches and experimental works on fiber bridging. Budiansky et al. [3], Hutchinson et al. [4], Cox et al. [5] and Bao et al. [6] have developed micromechanical models in order to describe fiber bridging. Other efforts in the development of geometry independent bridging laws have been conducted by Sørensen and Jacobsen [7] and other researchers [8,9,10,11,12,13,14]. Other works also include extensive experimental and numerical efforts to accurately predict and model the fiber bridging mechanism [15,16,17,18].

The optimization and integration of the mechanism in question for composite structures can be achieved through numerical simulations. However, the most commonly used bilinear cohesive zone modeling in composite joints cannot accurately model the fracture toughness increment due to the bridging. This is due to the fact that the T–SL in both loading directions only represents the mechanical separation effect of the matrix interface. Efforts have been made towards the integration of the fiber bridging phenomenon in numerical models. Afshar et al. [19] have developed a numerical model through the extended finite element method (XFEM) by simulating the effect of bridging in the interface through non-linear springs.

The most common numerical approaches found in the literature are based on a modified cohesive zone modeling technique, where the effect of both the matrix and fibers are superimposed in a final multilinear T–SL. Such a technique can be found in the work of Heidari-Rarani et al. [20], where the superposition of two CZMs, representing the matrix interface and fiber bridging, respectively, is performed. The resulting T–SL follows a tri-linear form emerging from the simultaneous effect of the two bi-linear CZMs peaking at the same displacement jump.

Gong et al. [21] presented a model in the same principle as described above; however, in this case the two bi-linear CZMs did not have the same peak. The bridging part of the T–SL was initiated when the interface failed completely, which also abides to the physical behavior of the phenomenon. According to the above study, the proposed model [22] was experimentally validated and simplification techniques for more straight forward law extraction were presented [23].

Although a considerable amount of work has been conducted on the numerical simulation of the fiber bridging phenomenon, the applications are limited only on thermoset matrix composites to the authors’ knowledge. Additionally, in the case of thermoplastic laminates, where joining techniques such as welding are available, there is potential for further study on joints, not only for delamination. Considering that, the present work focused on the development of such a model, incorporating fiber bridging effects in low melt PAEK matrix laminates loaded in mixed mode conditions. This paper includes the analytical, experimental, and numerical procedure for the determination of a modified cohesive zone model. The general objective of the work was to simulate the bridging behavior present in previously conducted tests on mixed-mode Single-Lap-Shear (SLS) specimens, where the simple bilinear law did not manage to accurately approach the results.

In comparison to previously conducted studies, this work incorporated the application and further validation of the modified traction–separation law for fiber bridging simulation in joined thermoplastic composites. A complete experimental characterization of the mixed mode bridging behavior was performed. The development of two new computational tools contributed both in the more straightforward experimental results’ extraction and the more practical implementation of the model in question. Overall, the developed model adequately predicted the experimental results and was proved efficient in simulating the fiber bridging phenomenon.

## 2. Theoretical: The Modified Traction–Separation Law

The incorporation of the fiber bridging mechanism into the cohesive material model was achieved through the employment of a tri-linear T–SL, recently proposed by Gong et al. [21]. This section briefly describes the principle and theory of this cohesive zone model.

**Figure 1:**
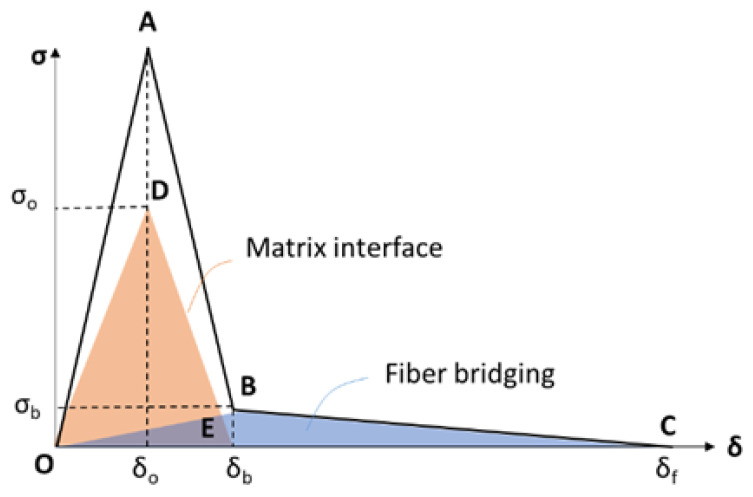
A schematic of the modified T–SL (ODE: Matrix interface T–SL; OBC: Fiber bridging T–SL; OABC: Combined trilinear T–SL).

The representation of the mechanical behavior of the interface is described by a tri-linear T–SL (OABC), which accrues from the superposition of two simple bi-linear CZMs (Figure 1). For the representation of the matrix interface separation, the bi-linear part (ODE) is employed. For the $0-\delta_{0}$ displacement jump of the cohesive, linear elastic behavior is prescribed with $K_{1}$ stiffness, followed by the degradation part (DE) of the law. In the same principle, the effect of fiber bridging is modeled with another bi-linear law (OBC), where the onset of its degradation coincides with the total failure of the matrix interface. Finally, by superimposing the described components, a tri-linear traction–separation law (OABC) is obtained [21].

The tri-linear CZM is described by the following equation:

(1) $$\sigma =(1-d)D_{ij}^{0}d$$

where the initial stiffness tensor is defined as

(2) $$D_{ij}^{0}={\bar{\delta }}_{ij}K_{0}$$

and the global damage variable is divided as follows:

(3) $$d=\{\begin{array}{c} 0,\delta \leq 0\\ \left(1-\frac{K_{AB}}{K_{0}}\right)\left(1-\frac{\delta_{0}}{\delta }\right),\delta_{0}<\delta \leq \delta_{b}\\ 1-\frac{K_{BC}}{K_{0}}\left(1-\frac{\delta_{f}}{\delta }\right),\delta_{b}<\delta \leq \delta_{f}\\ 1,\delta \geq \delta_{f} \end{array}$$

where $K_{0}$, $K_{AB}$ and $K_{BC}$ stand for the (*OA*), (*AB*) and (*BC*) lines’ tangents, respectively. Other crucial parameters required for the composition of the T–SL are the initial fracture toughness $G_{ini}$, the propagation fracture toughness $G_{prop}$, the interfacial strength $\sigma_{0}$, the bridging stress $\sigma_{b}$, as well as the stiffnesses $K_{1}$ (or $K_{OD}$) and $K_{2}$ (or $K_{OB}$). In the case of mixed-mode loading, each of these parameters needed to be defined both for mode I and mode II.

## 3. Overall Approach

The development of the final modified T–SL for the co-consolidated laminates consisted of experimental, analytical, and numerical parts. Initially, the required tests, mentioned in Section 2, were conducted; specifically, double cantilever beam (DCB) specimens’ tests for mode I and edge notched flexure (ENF) specimens’ tests for mode II. The data acquired from this first step are the fracture toughness values, as well as the resistance curves, which are indicative of the bridging behavior of the specimens. However, the latter could not be generalized to any coupon for the modeling of fiber bridging as they are geometry dependent. To overcome this issue, a bridging law for each loading mode needed to be defined.

The technique involved extracting the bridging law and, subsequently, the bridging stress $\sigma_{b}$ is described in [23] and comprises a simplified procedure for measuring the crack opening displacement (COD) in the DCB and the crack sliding displacement (CSD) in the ENF specimens, thus eliminating the need for complex and time-consuming digital image correlation monitoring techniques during testing. The *R*-curves extracted from the two mechanical tests were introduced in two FE models simulating the two tests, respectively. The critical energy release rate or fracture toughness values obeyed the *R*-curve behavior and the COD could be measured numerically. In this manner the bridging stress–crack propagation curve could be plotted using the following equation:

(4) $$\sigma_{b}=\frac{\partial G}{\partial \delta^{*}}$$

where $\delta^{*}$ stands for the crack opening or sliding displacement. With this last step, all the parameters needed to construct the trilinear T–SL became available.

Finally, two different options for the numerical implementation of the modified mixed-mode CZM were followed, including the analysis through an already available LS Dyna’s material model and the development of a user defined subroutine. The latter two methods were employed for the simulation of SLS specimens, and the results were compared and validated upon available test data. Furthermore, the numerical results that were obtained from the modified models were compared with the output of a simple bilinear T–SL model.

**Figure 2:**
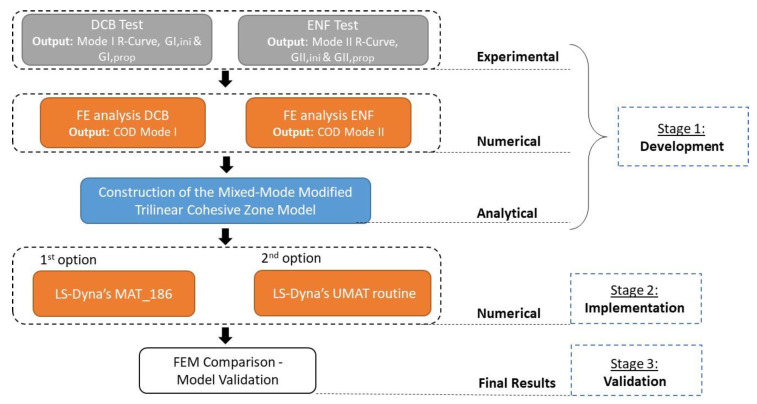
Modeling procedure flowchart.

Overall, the procedure described above was divided in three stages: development, implementation, and validation (Figure 2). In the first step, the mandatory experimental, numerical, and analytical data were acquired. Subsequently these were used as input in the numerical models of the implementation stage and finally, the results were compared and validated throughout the third stage. In the following sections, the three stages are analyzed in detail.

## 4. Stage 1: Development

### 4.1. Experimental

#### 4.1.1. Mode I—DCB

**Figure 3:**
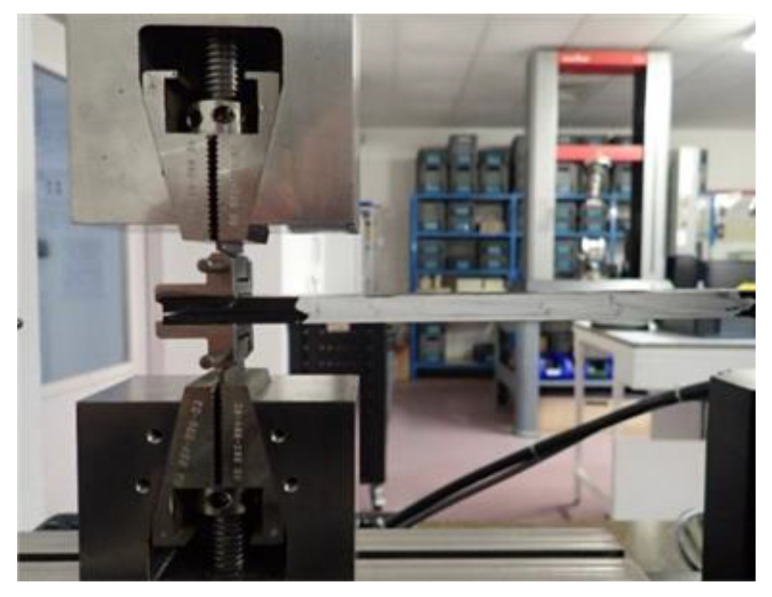
Mode I loading experimental set up.

The mechanical testing of laminates in mode I loading conditions was performed according to the ASTM D5528-01 standard [24,25,26]. A total of eight co-consolidated specimens were cut in 150 mm length, 25 mm width and 1.8 mm thickness for each substrate. The plies followed a quasi-isotropic stacking [0°/45°/90°/45°/−45°]s and the predefined crack was applied with a 63 mm Kapton film insert between the laminates. The prepreg material of the laminates was a LM-PAEK-based matrix, reinforced with TC1225 carbon fibers, provided by Toray. The experimental set-up of the mode I test is illustrated in Figure 3.

Piano hinge tabs were bonded to the edges of the cantilever beam in order to apply the opening load, according to the standard. The tests were held in room temperature conditions, prescribing a constant crosshead displacement rate of 1 mm/min. During the loading of the specimens, the crack propagation was optically monitored through a digital microscope.

The fracture toughness values for each state of the propagation were obtained through the modified beam theory method. Finally, the *R*-curves and the $G_{I,ini}$ and $G_{I,prop}$ properties of the specimens were computed.

**Figure 4:**
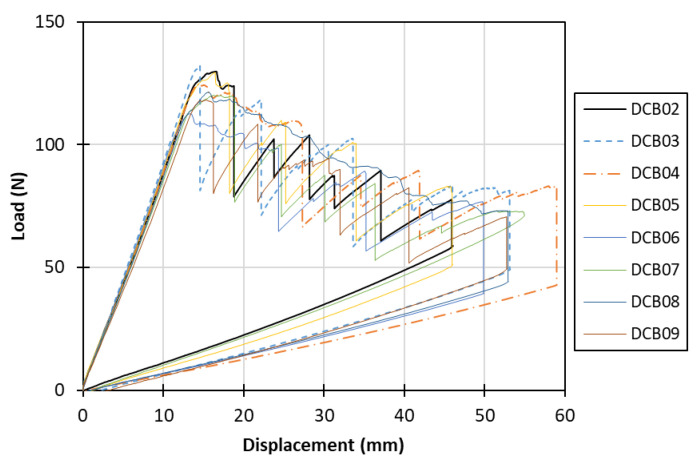
Mode I experimental load–displacement curves.

**Figure 5:**
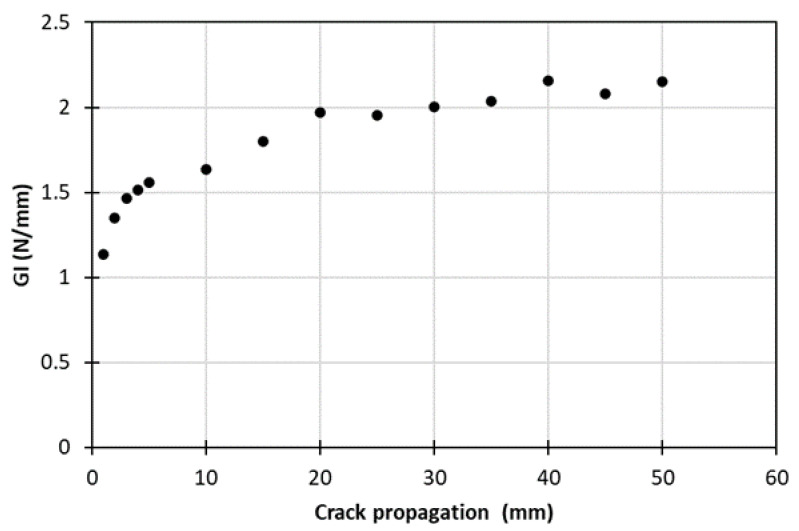
Characteristic *R*-curve extracted from Mode I tests.

The mechanical testing conducted on DCB specimens showed similar response and close crack initiation load values for all the specimens; indicatively, in Figure 4, the load–displacement curves for the eight specimens (DCB02–DCB09) are presented. Figure 5 depicts a characteristic *R*-curve for the co-consolidated laminates. The initiation and propagation fracture toughness values in this case were roughly 1.1 N/mm and 2.1 N/mm, respectively, while the length of the crack propagation for the stabilization of the fracture toughness to a steady value, also defined as the bridging length, was about 40 mm.

**Figure 6:**
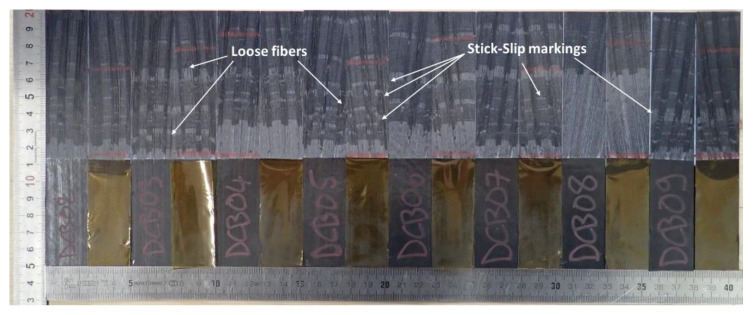
DCB specimens’ fractured surfaces after testing.

In Figure 6, the fractured surfaces along the DCB specimens are shown. It is obvious that the propagation of the crack suffered strong stick-slip behavior, which is noticed both from the color inhomogeneity of the surface and the load–displacement curves. Additionally, loose fibers and bridged fiber bundles can be found by a closer surface inspection, confirming the bridging behavior presented by the extracted *R*-Curve.

#### 4.1.2. Mode II—ENF

**Figure 7:**
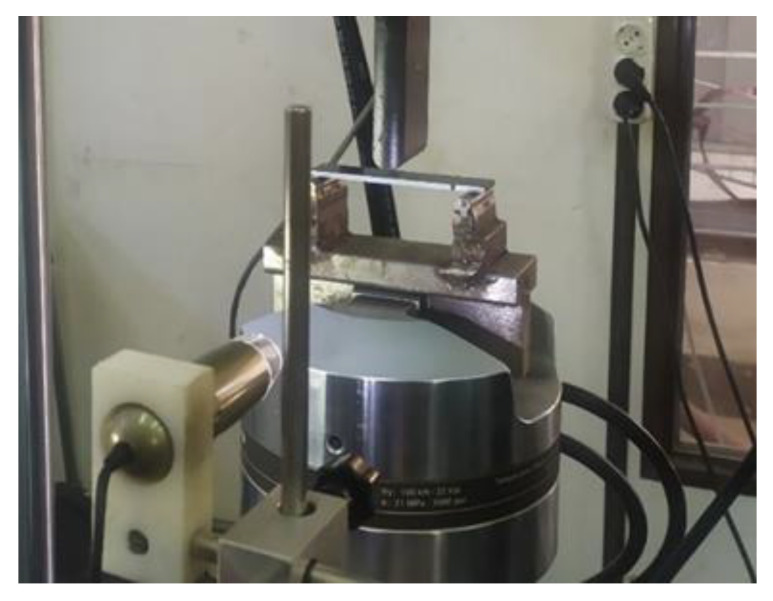
Mode II loading experimental configuration.

The mode II tests were performed according to the AITM 1.0006 standard [27,28,29]. Six specimens, with adherents of 115 mm by 25 mm dimensions and a 45 mm predefined cracking, were studied. In the same principle as mode I tests, the crack propagation under the constantly increasing load was monitored through a digital microscope [30]. The testing machine used was an MTS universal with a load cell capacity of 100 kN (Figure 7).

**Figure 8:**
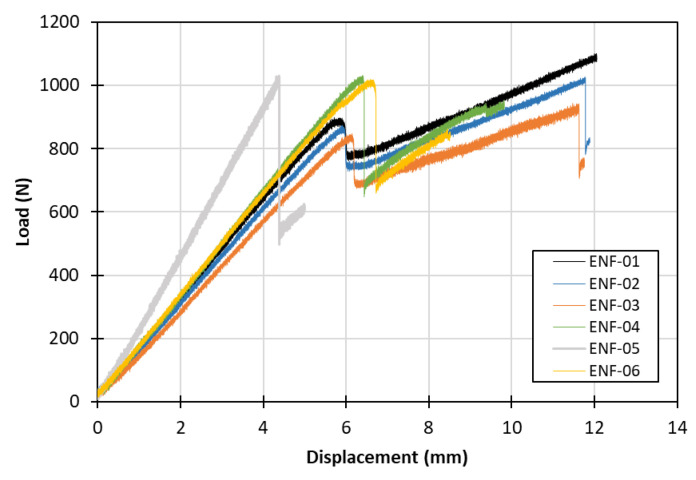
Mode II experimental load–displacement curves.

Figure 8 depicts the load–displacement response of the ENF specimens, as obtained from mode II testing. In this case, the ENF-05 specimen was excluded from the study, as a considerable difference was recorded due to remaining cohesion at the Kapton region and, subsequently, falsely increased stiffness and interlaminar strength. Specimens ENF-04 and ENF-06 also presented a slightly higher maximum load prior to failure, possibly for the same cause as ENF-05, although the discrepancy was considered acceptable.

**Figure 9:**
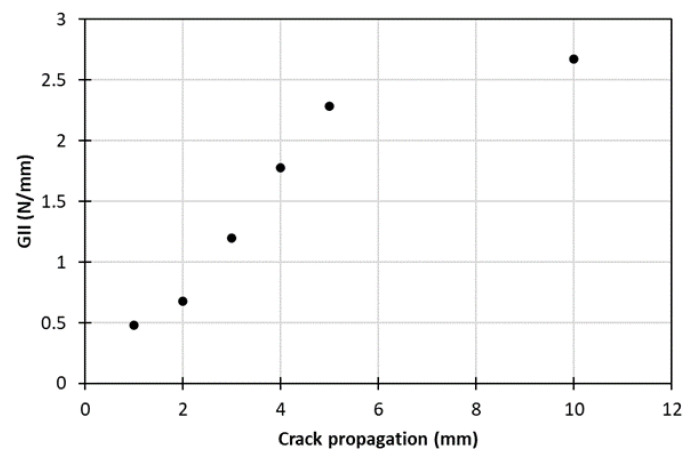
Characteristic *R*-curve extracted from Mode II tests.

Figure 9 shows the characteristic fracture toughness–crack propagation curve, where the initiation $G_{II}$ value was measured quite low, at about 0.5 N/mm, and the steady state $G_{II}$ value at 2.6 N/mm. For this test, the bridging length was considerably lower from the mode I test, lying at 5 mm. It is noteworthy that due to the nature of the three-point bending test, the load–crack propagated length data recording proved to be quite difficult, rendering the acquisition of more data points on the *R*-curve impossible.

**Figure 10:**
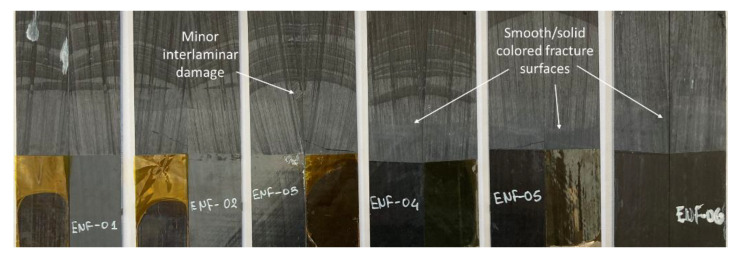
ENF specimens’ fractured surfaces after testing.

The fracture surfaces of the ENF specimens after testing, as shown in Figure 10, indicated a considerably lower amount of bridged and loose fibers, differing to the previous case. As expected, the crack stick-slip phenomenon was visually absent in mode II.

#### 4.1.3. Mixed Mode SLS

The mixed mode-loaded SLS specimens used for the implementation and validation stage of the model are briefly described. The experimental procedure was based on the ASTM 5868-01 standard. The stacking sequence of the composite laminates was [0°/−45°/45°/90°/45°/−45°]s, joined via co-consolidation at the overlapped area. The dimensions of the substrates were 101.6 mm in length, 25.4 mm wide and a total number of six specimens were tested. The coupons were loaded in a quasi-static displacement rate of 1 mm/min until final rupture.

### 4.2. Numerical Part

**Figure 11:**
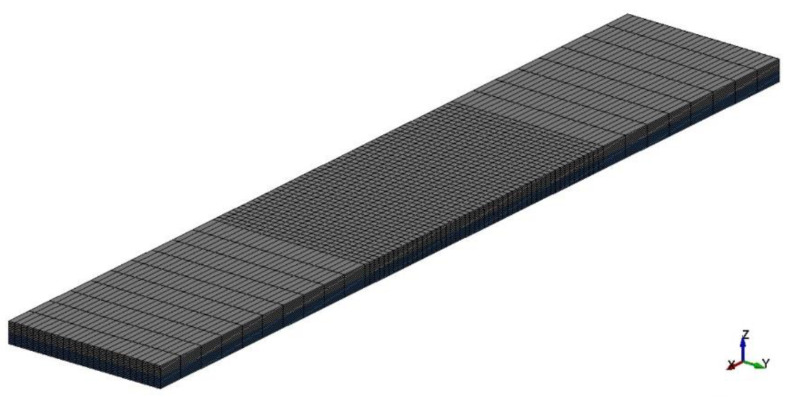
Typical FE mesh of the DCB coupon.

The numerical aspect of the present stage incorporates FE simulations for the numerical estimation of the bridging law in DCB and ENF specimens. The numerical analyses were performed using the commercial FE suite, LS Dyna, using 8-noded reduced integrated solid elements for the composite laminates (Figure 11) [31,32,33].

#### User Defined Subroutine—UMAT_RCurve

The numerical part also includes the development of the user defined routines in order to introduce the experimental *R*-curves in the DCB and ENF models (UMAT_*R*Curve). Simulations were performed in LS Dyna, while the proprietary scripting was written in FORTRAN 77 programming language and compiled through Intel Visual Fortran Compiler 2010. The developed subroutine was implemented through the LS Dyna’s available material model UMAT43c, which is suitable for modeling three dimensional cohesive elements [34,35,36].

The mechanical response of the user defined model was exactly the same as a simple bi-linear CZM, with the exception that the fracture toughness in each loading mode was not a constant value but was position-dependent, based on the experimental *R*-curves. In order to implement this, a cohesive element position reading algorithm had to be written.

**Figure 12:**
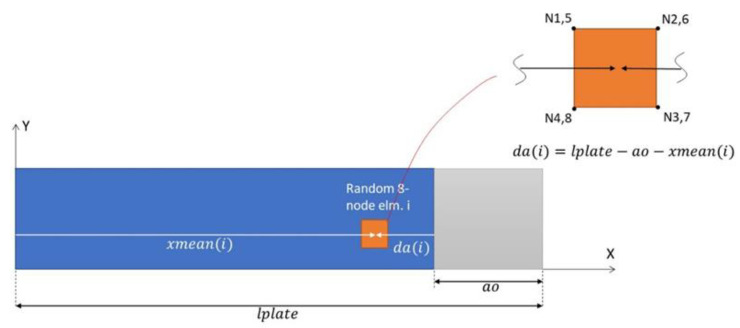
Element position calculation principle for the UMAT_*R*Curve routine.

As an input, the total coupon length (lplate) and the pre-cracked length ($a_{0}$) were required. Through an iterative procedure, the nodal coordinates of the cohesive elements were obtained and, subsequently, the distance from the element’s center to the crack tip (da(i)) was calculated (Figure 12). By these means, the fracture toughness for each cohesive element was prescribed from the *R*-curve, which also constitutes a subroutine’s input.

**Figure 13:**
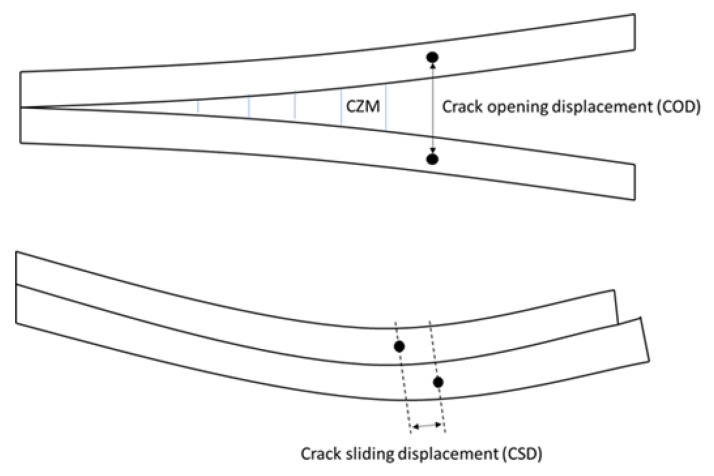
Measurement of the COD and CSD from DCB and ENF specimens, respectively.

The objective of this numerical step was to bypass the need for complex experimental monitoring techniques for the crack opening displacement (COD) and crack sliding displacement (CSD) (Figure 13) by directly extracting the desired curve through FE models of the DCB and ENF specimens. This procedure has been proposed in [23]; however, it has only been applied for mode I loading.

**Figure 14:**
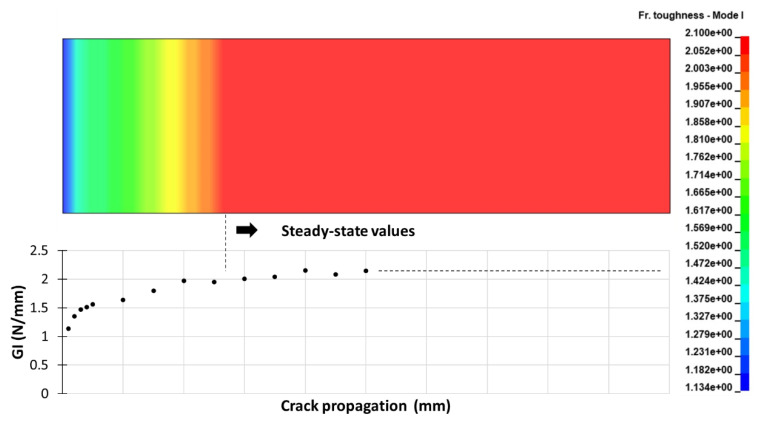
Mode I fracture toughness distribution at the DCB coupon’s cohesive interface (UMAT_*R*Curve).

Based on the procedure described above, the fracture toughness distribution along the cohesive elements at the interface of the DCB is shown in Figure 14. The lowest *G* value was applied at the crack-front elements, increasing according to the experimentally defined *R*-curve. Similarly, the same principle was applied to the ENF cohesive interface.

**Figure 15:**
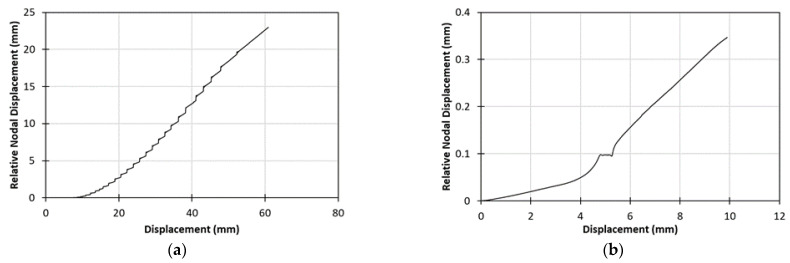
(**a**) Numerically computed COD—displacement curve for the DCB specimen; (**b**) Numerically computed CSD—crosshead displacement curve—for the ENF specimen.

The COD and CSD computed from the two numerical analyses are presented in Figure 15. In both cases, the relative nodal displacements between the joined laminates were recorded for each timestep, in the appropriate directions for each loading case, in relation to the modeled crosshead displacement, imposed by the boundary conditions. The curves showed expected behavior both for mode I and mode II, as indicated by previous studies [23,37,38,39]. In Figure 15a, the relative nodal displacement reached a maximum value of about 23 mm, following a slightly parabolic increase. In Figure 15b, the relative nodal displacement had a maximum relative nodal displacement of 0.35 mm, presenting a plateau at the crack initiation point.

### 4.3. Analytical Part

**Figure 16:**
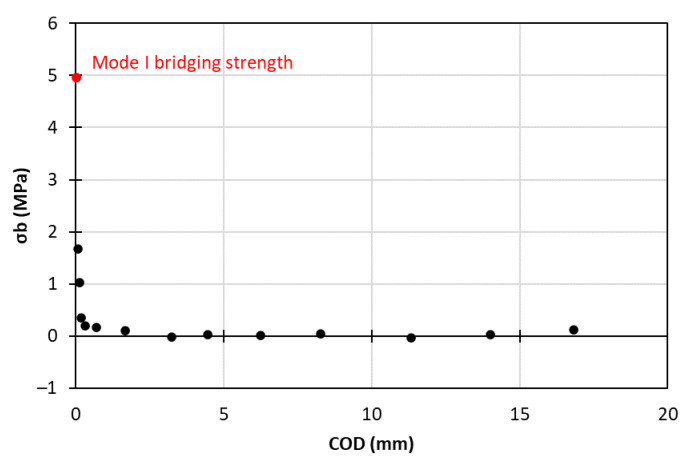
Mode I bridging stress—COD curve.

**Figure 17:**
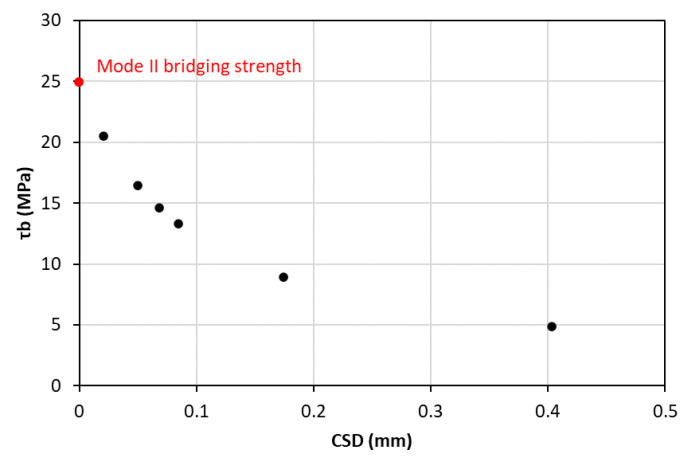
Mode II bridging stress—CSD curve.

In this subsection, the calculations for the mixed-mode tri-linear T–SL are presented. As the first requirement, the bridging stress could be calculated from the differentiation of the fracture toughness with respect to the COD or the CSD, depending on the loading mode (Equation (4)). In this way, the bridging stress–opening/sliding displacement curves could be drawn, as shown in Figure 16 and Figure 17. The first points in these two diagrams corresponded to mode I and II bridging strength, specifically $\sigma_{I,b}=4.96\;\mathrm{MPa}$ and $\sigma_{II,b}=\tau_{b}=22.4\;\mathrm{MPa}$.

According to the literature [21], the peak traction of the matrix interface contributions ($\sigma_{0,I}$, $\sigma_{0,II}$) can be approximated by a percentage of 55–65% of the interfacial matrix strength in normal and shearing directions, respectively. In this case, a 0.65 ratio was selected, resulting in:

(5) $$\sigma_{0,I}=0.65T=55.9\;\mathrm{MPa}$$

(6) $$\sigma_{0,II}=0.65S=27.3\;\mathrm{MPa}$$

$K_{1}$ and $K_{2}$ describe the stiffnesses for the two superimposed T–SLs to the corresponding loading mode. The $K_{1}$ value can be calculated by the following equation

(7) $$K_{1}=\frac{2E_{Z}}{h}$$

where $E_{Z}$ was the through the thickness laminates’ stiffness, and h half of the DCB or ENF specimen thickness. $K_{2}$ was defined through simple trigonometric calculations from the T–SL schematic, resulting in $K_{1}=9.136\;\mathrm{MPa}$ and $K_{2}=0.143\;\mathrm{MPa}$.

Furthermore, the displacement jumps for each loading mode could be calculated in the following manner:

(8) $$\delta_{0,I}=\frac{\sigma_{0,I}}{K_{1,I}}=0.006\;\mathrm{mm}$$

(9) $$\delta_{b,I}=\frac{2G_{I,ini}}{\sigma_{0,I}}=0.04\;\mathrm{mm}$$

(10) $$\delta_{f,I}=\frac{2\left(G_{I,prop}-G_{I,ini}\right)}{\sigma_{b,I}}=0.389\;\mathrm{mm}$$

**Figure 18:**
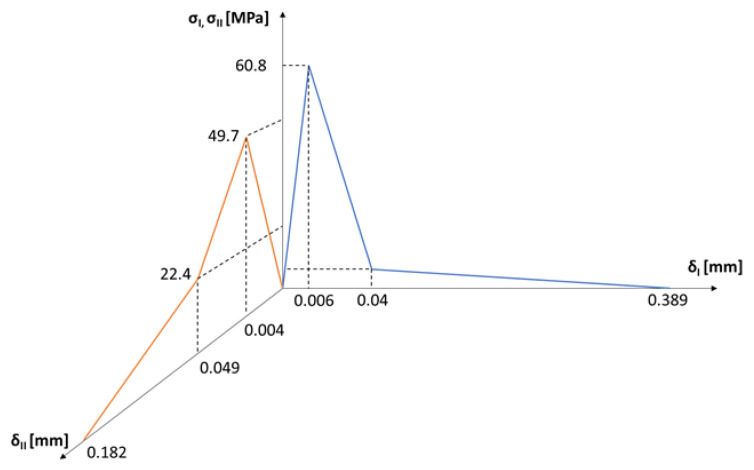
The final mixed-mode tri-linear T–SL.

Following the same procedure for mode II loading, $\delta_{0,II}$, $\delta_{b,II}$ and $\delta_{f,II}$ could be calculated as 0.004 mm, 0.049 mm, and 0.182 mm, respectively. Ultimately, the final form of the mixed-mode tri-linear T–SL is shown in Figure 18.

## 5. Stage 2: Implementation

Numerical analyses were held in this stage to determine the behavior for the co-consolidated laminates under mixed mode loading. The simple bilinear T–SL was implemented through the LS Dyna’s MAT_138 material model [40]. The numerical implementation of the finally developed model of the modified tri-linear T–SL was studied in two ways.

### 5.1. User Defined Subroutine—UMAT_Tril

**Figure 19:**
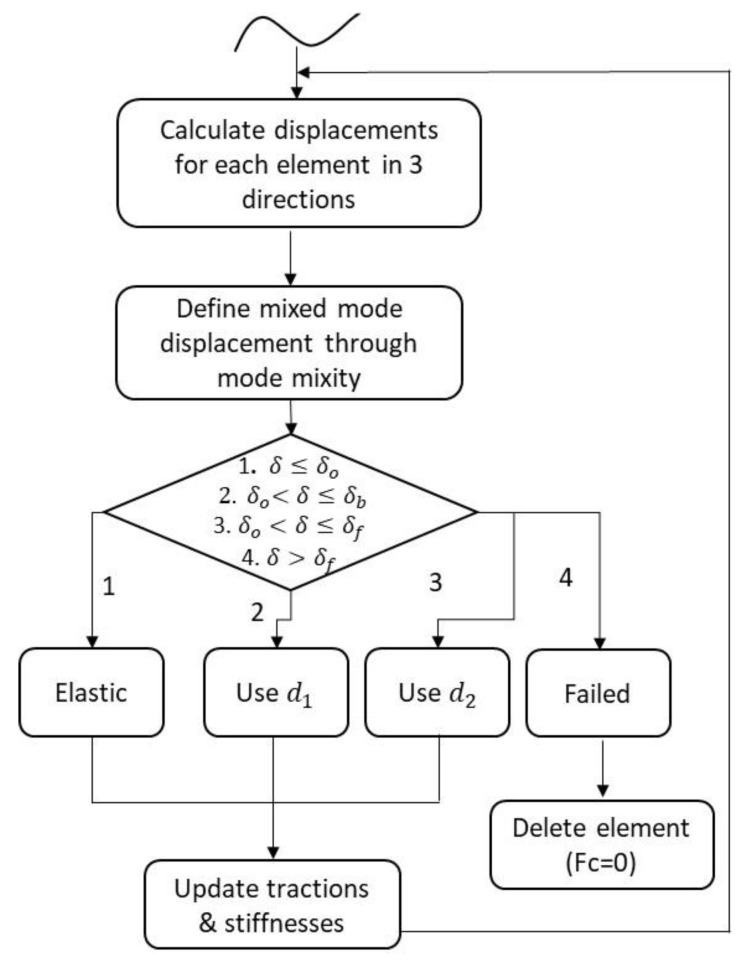
UMAT_Tril subroutine flowchart.

The numerical application of the final modified T–SL required the development of another subroutine, able to follow the trilinear traction–separation behavior of the cohesive elements. The developed subroutine, UMAT_Tril, required the user input data previously calculated from the experimental, numerical, and analytical procedures, namely the bridging strength, the interfacial strength, the initiation, and propagation fracture toughness values, the initial stiffnesses of the matrix and fiber bridging elastic response. These parameters needed to be defined prior to the analysis, both for mode I and II loading conditions, as the routine calculates the mixed-mode displacements and stresses of the cohesive elements. In Figure 19, the flowchart of the iterative calculation procedure for every element in UMAT_Tril is depicted.

Based on the element’s mixed-mode displacement for each step, the algorithm selected its status: 1. elastic response; 2. matrix interface degradation; 3. fiber bridging contribution; 4. total failure. Each damage variable indicated the degradation percentage of the corresponding modeled part. The two main damage variables *d*_1_ and *d*_2_, corresponding to the matrix interface and bridging effect degradation, respectively, were constantly available throughout the computational procedure. This enabled the definition of the damage status for every cohesive element in each time step of the simulation.

### 5.2. LS Dyna’s MAT_186

**Figure 20:**
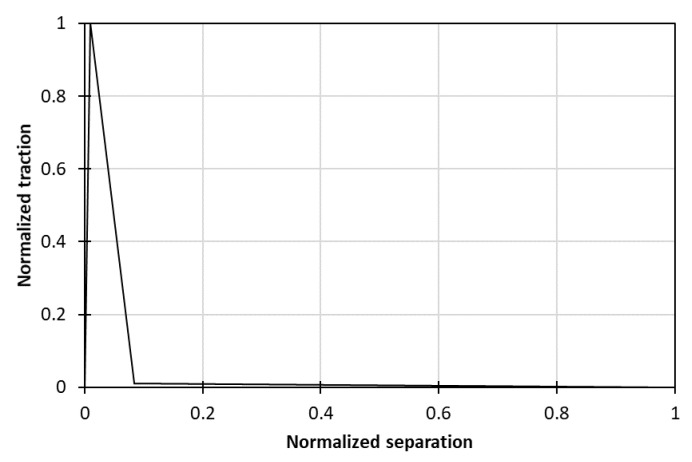
Normalized traction-separation curve used in MAT_186 material model.

Another implementation technique followed for the application of the tri-linear T–SL was through the employment of LS Dyna’s MAT_186 (MAT_COHESIVE_GENERAL) material model, which allowed the definition of user-defined T–SL by prescribing a normalized curve (Figure 20). Different cohesive responses were prescribed for the two loading modes, thus making the mixed-mode simulation possible. The previously developed subroutine UMAT_Tril worked in the exact same computational way as MAT_186, so identical results were to be expected.

## 6. Stage 3: Final Results and Validation

In this section, the final mechanical responses of the mixed-mode SLS specimens from the numerical simulations are presented. The numerical results included the output data for the trilinear T–SL modeled interfaces, and then they were compared to the bilinear modeled interface response and experimentally acquired data.

**Figure 21:**
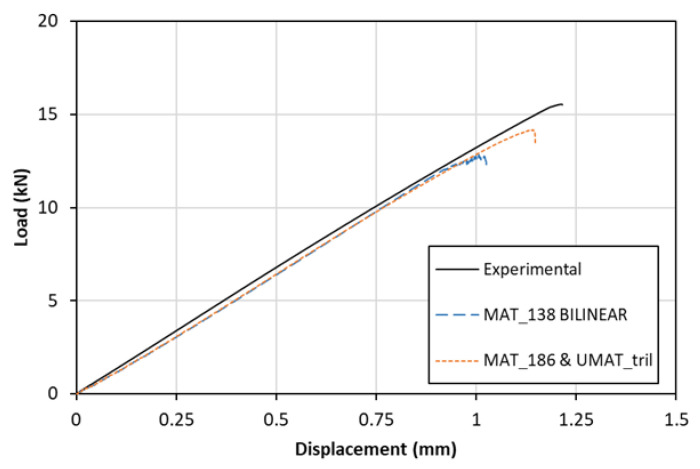
Experimental and numerical load–displacement curves of the SLS specimen.

**Figure 22:**
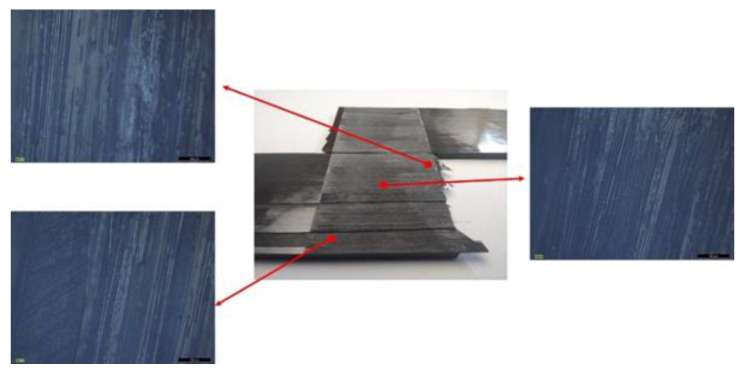
SLS specimens’ characteristic fractured surface investigated through optical microscopy.

Figure 21 depicts the load displacement curves computed numerically. The experimentally obtained curve peaked at 16 MPa prior to failure. Significantly lower was the maximum load for the bilinearly modeled coupon. Such a difference was expected, as MAT_138 did not reckon in the fiber bridging effect and was extensively present on the failed areas of the tested SLS specimens (Figure 22). The mechanical response of the two numerical models (MAT_186 & UMAT_Tril) was virtually the same due to their identical computational behavior. The models based on the modified traction–separation law managed to approach the experimental behavior more accurately than the simple bilinear model. Although the load–displacement curves did not fully capture the real-world mechanical behavior, considerable improvement occurred.

**Figure 23:**
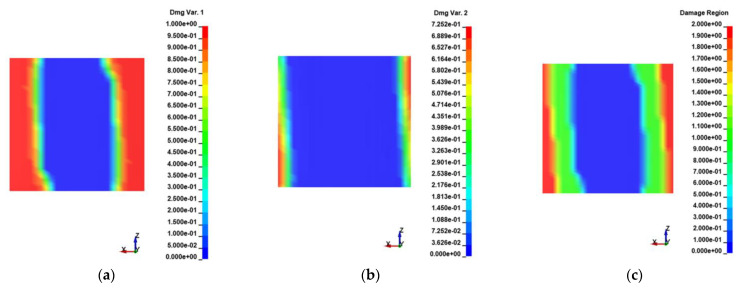
(**a**) *d*_1_ damage variable and (**b**) *d*_2_ damage variable monitored through UMAT_Tril analysis; (**c**) CZM degradation region (0: elastic region; 1: matrix interface degradation; 2: fiber bridging degradation).

The damage variables *d*_1_ and *d*_2_ obtained from the UMAT_Tril simulation for the final timestep prior to the failure initiation are plotted in Figure 23a,b. A damage region contour was also captured (Figure 23c), indicating each element’s degradation region. Such a result led to the conclusion that the developed models presented quite an accurate representation of the complex fiber-bridging phenomenon, as the damage progression and failure of the tested specimens showed a similar pattern by examining the imprints of the fractured surfaces (Figure 22).

## 7. Conclusions

In the present work, a model for simulating fiber bridging along the co-consolidated interface of thermoplastic laminates was developed, based on a modified trilinear T–SL.

The conclusions emerging from the study are listed below:

- Overall, the procedure described above was proven adequately accurate for simulating the fiber bridging behavior of mixed mode-loaded co-consolidated thermoplastic laminates. Although the numerical results were not totally identical with the mechanical tests’ data, the improvement over the traditional bilinear cohesive zone modeling was quite considerable. The trilinear T–SL managed to simulate the increased fracture properties due to fiber bridging, following the mechanical aspects of the phenomenon.
- The developed subroutine, UMAT_*R*Curve, proved to be an efficient way to numerically measure the crack opening/sliding displacements in both loading modes. A significant amount of experimental work is omitted and maintained though the validity and accuracy of the results.
- In the final numerical step, where two implementation options were studied, the output was the same. However, in the case of the UMAT_Tril subroutine, additional flexibility in terms of results’ monitoring was given. The status of every cohesive element’s degradation could be recorded for each timestep, offering the opportunity for meticulous CZM behavior observation during failure.

Finally, it is evident that such a procedure added complexity to the numerical simulation when compared to the mostly used bilinear T–SL. With this in mind, this technique should be used in cases where the fiber bridging phenomenon is prominent, noticeably affecting the mechanical response of the joint.

Apart from the application on co-consolidated joints, this technique is feasible to be applied in any other type of joint for thermoplastic laminates. Thermoplastic welding, for example, constituting a fusion procedure, usually without the integration of third material into the joint, is highly likely to develop the ideal conditions for extensive bridging of the fibers.

## Data Availability

Not applicable.
